# Challenges and adaptations in pancreatic cancer surgery during the COVID-19 pandemic in a high-volume center

**DOI:** 10.1186/s12885-025-13512-6

**Published:** 2025-01-29

**Authors:** Mohammed Al-Saeedi, Ali Ramouz, Elias Khajeh, Sakher Shraim, Alexander Werba, Georgios Polychronidis, Arianeb Mehrabi, Martin Loos

**Affiliations:** 1 Department of General, Visceral, and Transplantation Surgery, University of Heidelberg, Heidelberg, Germany; 2 Division of Surgical Oncology, Department of General, Visceral, and Transplant Surgery, Ruprecht Karls University Heidelberg, Im Neuenheimer Feld 420, 69120 Heidelberg, Germany

**Keywords:** Pancreas cancer, Pancreatoduodenectomy, COVID-19, Mortality, Standardization

## Abstract

**Background:**

The COVID-19 pandemic affected healthcare systems worldwide, disrupting elective surgeries including those for cancer treatment. This study examines the effects of the pandemic on outcomes of pancreatic cancer surgeries at a specialized high-volume surgery center.

**Materials and methods:**

This study compared surgical volume and outcomes of pancreas resections between the pre-pandemic (January 2019 to February 2020), early pandemic (March 2020 to January 2021), and late pandemic (February 2021 to December 2021) periods. Perioperative and postoperative data were retrospectively analyzed from a prospectively maintained database together with surgical complications, mortality rates, and hospital stays.

**Results:**

There was no significant reduction in the number of pancreas resections performed during the pandemic. The rate of primary resectable tumors was significantly lower during the late pandemic phase (66% vs. 65.9% vs. 56.5%; *P* = 0.024), and subsequently application of neoadjuvant therapies increased in the late pandemic phase (26% vs. 25.4% vs. 33.8%; *P* = 0.079). The number of chemotherapy cycles were also higher during the late pandemic phase (*P* = 0.009). Surgical complication rates were higher during the late pandemic phase (47.8% vs. 45.6% vs. 56%; *P* = 0.043), but mortality rates remained low (30-day mortality: 1.6% vs. 1% vs. 3.7%, *P* = 0.116; 90-day mortality: 2.5% vs. 1.6% vs. 3.7%, *P* = 0.296).

**Conclusion:**

Our results indicate effective management of pancreatic cancer despite the challenges presented by the pandemic. These findings suggest that centralized, specialized surgical centers can maintain high-quality care of patients with pancreatic cancer during crises like the COVID-19 pandemic. These findings underscore the importance of timely surgical interventions for cancer patients, even when the healthcare system is disrupted.

## Introduction

COVID-19, the disease caused by severe acute respiratory syndrome coronavirus 2 (SARS-CoV-2), was declared a global pandemic by the World Health Organization (WHO) in March 2020. Since then, the pandemic has significantly disrupted the treatment of non-infectious diseases, particularly malignancies [1]. Considering the high rate of infection and mortality induced by SARS-CoV-2, severe burden was imposed to the health care systems all around the world [2, 3]. This burden resulted in considerable limitations in capacity of the hospital and intensive care units (ICU) [3, 4]. Accordingly, these limitations impacted the elective surgeries and restricted the prioritization of the cancer patients considering the available resources. According to the WHO, disease management was disrupted in 42% of patients with non-infectious diseases [1, 4] and surgical care was delayed in 76% of patients with cancer [1]. Despite this, cancer-related mortality has remained consistent.

Pancreatic cancer is a particularly aggressive disease, with poor long-term outcomes, particularly among patients with pancreatic ductal adenocarcinoma (PDAC). The main reasons for this poor prognosis are late detection, low oncological response, and early dissemination. In addition to this, delayed treatment can exacerbate the oncological and overall survival of patients even further [5, 6]. Therefore, the treatment delays caused by the COVID-19 pandemic may have posed more of a threat to these patients [7]. Indeed, the number of pancreas resections decreased more than two-fold during the pandemic [8]. Up-front surgeries were further disrupted by reductions in the number of inpatient beds, outpatient visits, and diagnostic procedures [9–11].

Our center is specialized in hepatopancreatobiliary (HPB) surgery and performs a high number of pancreas surgeries each year. In the current study, the impact of the pandemic on the number and outcomes of pancreas resections for PDAC by retrospectively analyzing data from a prospectively maintained database was evaluated.

## Methods

### Study population

All patients who underwent pancreas resection for malignancies between January 2019 and December 2022 were included. Peri- and postoperative data were collected from our prospectively maintained database. Approval of the study was granted by the independent ethics committee (institutional review board) of the University of Heidelberg (approval number: S-011/2015). The ethics committee waived the consent to participation because of the retrospective nature of the study. All procedures were performed according to the most recent revision of the Declaration of Helsinki and the standard operating procedure (SOP) of the division of pancreas surgery, which allows individualized decision-making and treatment for patients with pancreas tumors. Therapeutic decisions were made by an interdisciplinary institutional tumor board including surgeons, oncologists, radiologists, gastroenterologists, radiooncologists, and pathologists. The main characteristics of the SOP were a standardized surgical approach and precise postoperative care.

There were three patient groups – pre-pandemic, early pandemic, and late pandemic – depending on when the patient underwent pancreas resection. Patients who had surgery between January 2019 and February 2020 were in the pre-pandemic control group, patients who had surgery between March 2020 and January 2021 were in the early pandemic group, and patients who had surgery between February 2021 and December 2021 were in the late pandemic group. The early pandemic period marked the first wave of infections, and the late pandemic period marked the beginning of the vaccination campaign.

### Patient data collection

#### Pre- and intraoperative evaluations

Preoperative clinical data were recorded, including demographic data such as age and gender, preoperative neoadjuvant therapy, and type of pancreas malignancy.Therapy regimen and number of cycles were also obtained for patients with PDAC. The resectability status of the pancreatic cancer was defined based on the criteria introduced in National Comprehensive Cancer Network (NCCN) Version 3.2024 [12]. Data on the duration of the operation and amount of blood loss were also collected. The rate and amount of fresh frozen plasma and red blood cell transfusion during the patient’s hospital stay were recorded. The type of pancreas resection was categorized into three subgroups: pancreatoduodenectomy (PD), total pancreatectomy (TP), and distal pancreatectomy (DP). The difficulty of the PD and TP procedures were graded as standard procedures, additional venous resection, multivisceral resection, and additional arterial resection [13, 14].

#### Postoperative evaluations and follow-up

The overall rate of surgical complications was assessed independently of the severity grade. Delayed gastric emptying (DGE) [15], postoperative pancreas fistula (POPF) [16], and postpancreatectomy hemorrhage (PPH) [17] were also assessed and classified using the definition of the International Study Group of Pancreas Surgery. Relaparatomy was defined as further surgery because of complications after the primary pancreas resection. The duration of intermediate care (IMC), ICU stay, and hospital stay were also recorded. Postoperative mortality was defined as all-cause death occurring within 30 days and 90 days after surgery.

### Statistical analysis

Statistical analysis was performed using IBM SPSS Statistics for Windows, Version 27.0 (IBM Corp, released 2013, Armonk, NY). Categorical data were presented as frequencies and proportions and continuous data were presented as medians and ranges. Categorical data were compared using the Chi-square test of association or Fisher’s exact test. Differences in preoperative and postoperative continuous data between patients were determined using the Kruskal–Wallis test. A two-sided *P* value less than 0.05 was considered significant in all analyses.

To enable a comprehensive comparison across three timeframes, risk-adjusted outcomes were analyzed for the cohorts. A generalized estimating equation (GEE) model with a logit link function was used, accounting for the clustering of patients within each timeframe. The model included various preoperative and intraoperative patient characteristics, such as age, gender, ASA classification, administration of neoadjuvant therapy, type of surgery and tumor entity. The independent impact of the surgery timeframe on postoperative outcomes was then evaluated by incorporating the timepoints into the model. This allowed for the calculation of risk-adjusted odds ratios and expected rates of key outcomes, including surgical complications and 30- and 90-day mortality. All analyses were conducted using Stata Statistical Software for Mac, Release 17 (StataCorp LLC, 2021, College Station, TX).

## Results

### Demographic and preoperative data

Between January 2019 and December 2022, 871 patients underwent pancreas resection for malignancies in our center. The demographic data of the study cohort are summarized in Table 1. The most common indication for resection was PDAC (791 patients; 90.8%). The median patient age was 67.5 years (range: 34–90) and 465 (53.4%) patients were male. Neoadjuvant chemotherapy was performed in 243 (27.9%) patients. Of the included patients, 427 (49.0%) underwent PD, 255 (29.3%) underwent DP, and 189 (21.7%) underwent TP.

**Table 1:** Characteristics of patients undergoing pancreas surgery in 2019–2022

		Total (*N* = 871)	Time			*P* value
			Pre-pandemic phase (*N* = 320)	Early pandemic phase (*N* = 309)	Late pandemic phase (*N* = 242)	
Age		67.5 (34–90)	67 (37–87)	69 (35–87)	67 (34–90)	0.220
Gender	Male	465 (53.4%)	145 (45.3%)	157 (50.8%)	104 (43.0%)	0.158
	Female	406 (46.6%)	175 (54.7%)	152 (49.2%)	138 (57.0%)	
ASA	I	73 (8.4%)	19 (5.9%)	33 (10.7%)	21 (8.7%)	0.056
	II	445 (51.1%)	154 (48.1%)	165 (53.4%)	126 (52.1%)	
	III	353 (40.5%)	147 (45.9%)	111 (35.9%)	95 (39.3%)	
Diagnosis	PDAC	791 (90.8%)	288 (90.0%)	287 (92.9%)	216 (89.3%)	0.281
	Others	80 (9.2%)	32 (10.0%)	22 (7.1%)	26 (10.7%)	
Neoadjuvant chemotherapy		243 (27.9%)	84 (26.3%)	75 (24.3%)	84 (34.7%)	**0.018**
Procedure	PD	427 (49.0%)	166 (51.9%)	136 (44.0%)	125 (51.7%)	**0.025**
	TP	189 (21.7%)	78 (24.4%)	65 (21.0%)	46 (19.0%)	
	DP	255 (29.3%)	76 (23.8%)	108 (35.0%)	71 (29.3%)	
Procedure difficulty						
PD	Standard	234 (54.8%)	87 (52.4%)	78 (57.4%)	69 (55.2%)	0.207
	Venous resection	112 (26.2%)	48 (28.9%)	31 (22.8%)	33 (26.4%)	
	Multivisceral resection	62 (14.5%)	26 (15.7%)	26 (11.8%)	20 (16.3%)	
	Arterial resection	19 (4.4%)	5 (3.0%)	11 (8.1%)	3 (2.4%)	
TP	Standard	55 (29.1%)	22 (28.2%)	26 (40.0%)	7 (15.2%)	0.077
	Venous resection	40 (21.2%)	19 (24.4%)	10 (15.4%)	11 (23.9%)	
	Multivisceral resection	59 (31.2%)	24 (30.8%)	15 (23.1%)	20 (43.5%)	
	Arterial resection	35 (18.5%)	13 (16.7%)	14 (21.5%)	8 (17.4%)	
Operation time (min)		347.5 (110–877)	347.5 (117–640)	348 (110–877)	345.5 (117–662)	0.944
Blood loss (mL)		1050 (0–4800)	1100 (0–4800)	1000 (0–4700)	1000 (0–3800)	0.360
Morbidity		429 (49.3%)	153 (47.8%)	141 (45.6%)	135 (56.0%)	**0.043**
Clinically relevant DGE		29 (3.3%)	16 (5.0%)	8 (2.6%)	5 (2.1%)	0.105
Clinically relevant POPF*		41 (6.0%)	11 (4.5%)	12 (4.9%)	18 (9.2%)	0.085
Clinically relevant PPH		22 (2.5%)	11 (3.9%)	4 (1.2%)	7 (2.7%)	0.108
Relaparotomy		123 (14.1%)	51 (16.2%)	42 (13.7%)	30 (12.9%)	0.513
30-day mortality		16 (1.8%)	5 (1.6%)	3 (1.0%)	8 (3.3%)	0.116
90-day mortality		22 (2.5%)	8 (2.5%)	5 (1.6%)	9 (3.7%)	0.296
ICU stay		2 (1–82)	2 (1–82)	2 (1–60)	2 (1–30)	0.053
IMC stay		4 (1–87)	4 (1–83)	5 (1–87)	5 (2–42)	0.930
Hospital stay		14 (2–196)	14 (6–196)	14 (6–104)	15 (2–126)	0.497

Results are presented as median (range) or as *n* (%)

ASA: American Society of Anesthesiologists Physical Status; DGE: delayed gastric emptying; DP: distal pancreatectomy; ICU: intensive care unit; IMC: intermediate care; PD: pancreatoduodenectomy; PDAC: pancreatic ductal adenocarcinoma; POPF: postoperative pancreas fistula; PPH: postpancreatectomy hemorrhage; TP: total pancreatectomy

**Fig. 1:**
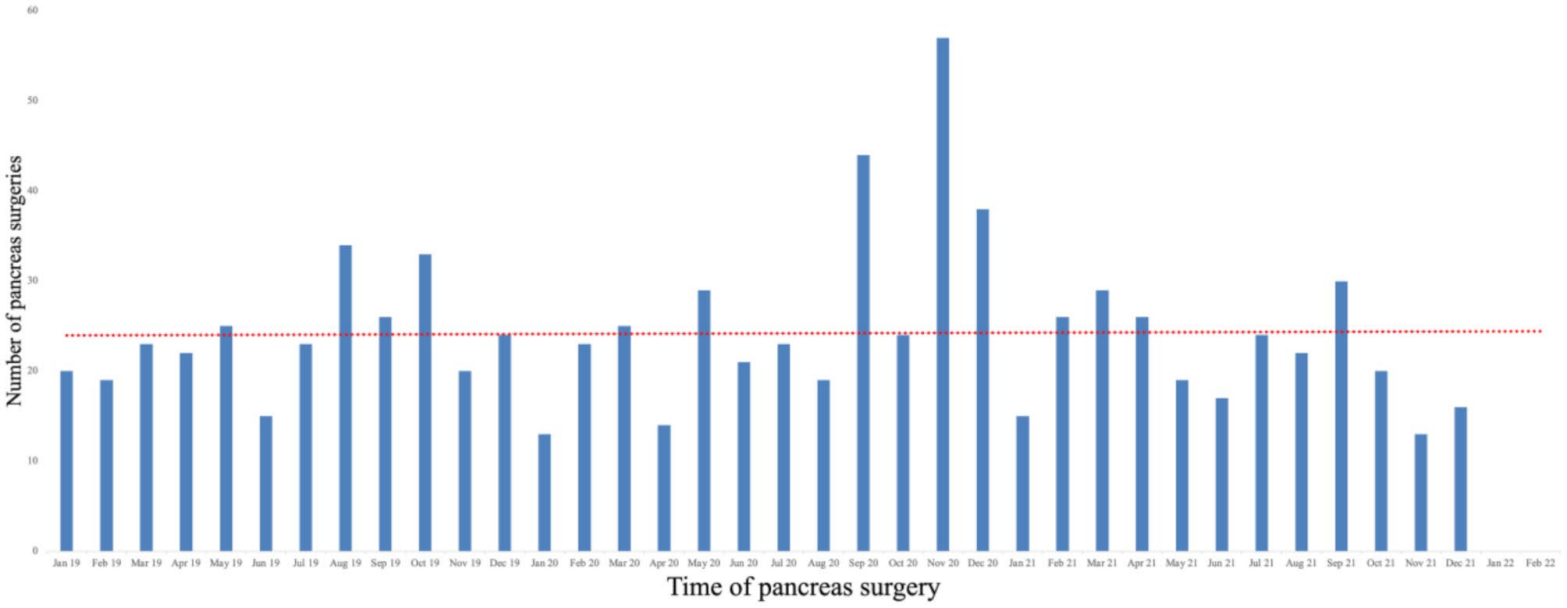
Timeline of the study period and effects of the COVID-19 pandemic on the frequency of pancreas resection in our clinic

The number of patients undergoing pancreas resection did not change significantly between the three time periods studied (Fig. 1). Linear prediction revealed a steady workflow of pancreas surgeries in our clinic. The number of pancreas surgeries increased by 5.2% during the early phase of the pandemic compared with the pre-pandemic phase, and the number of pancreas surgeries was lowest during the late phase of the pandemic, but these differences were not significant (Fig. 1).

**Fig. 2:**
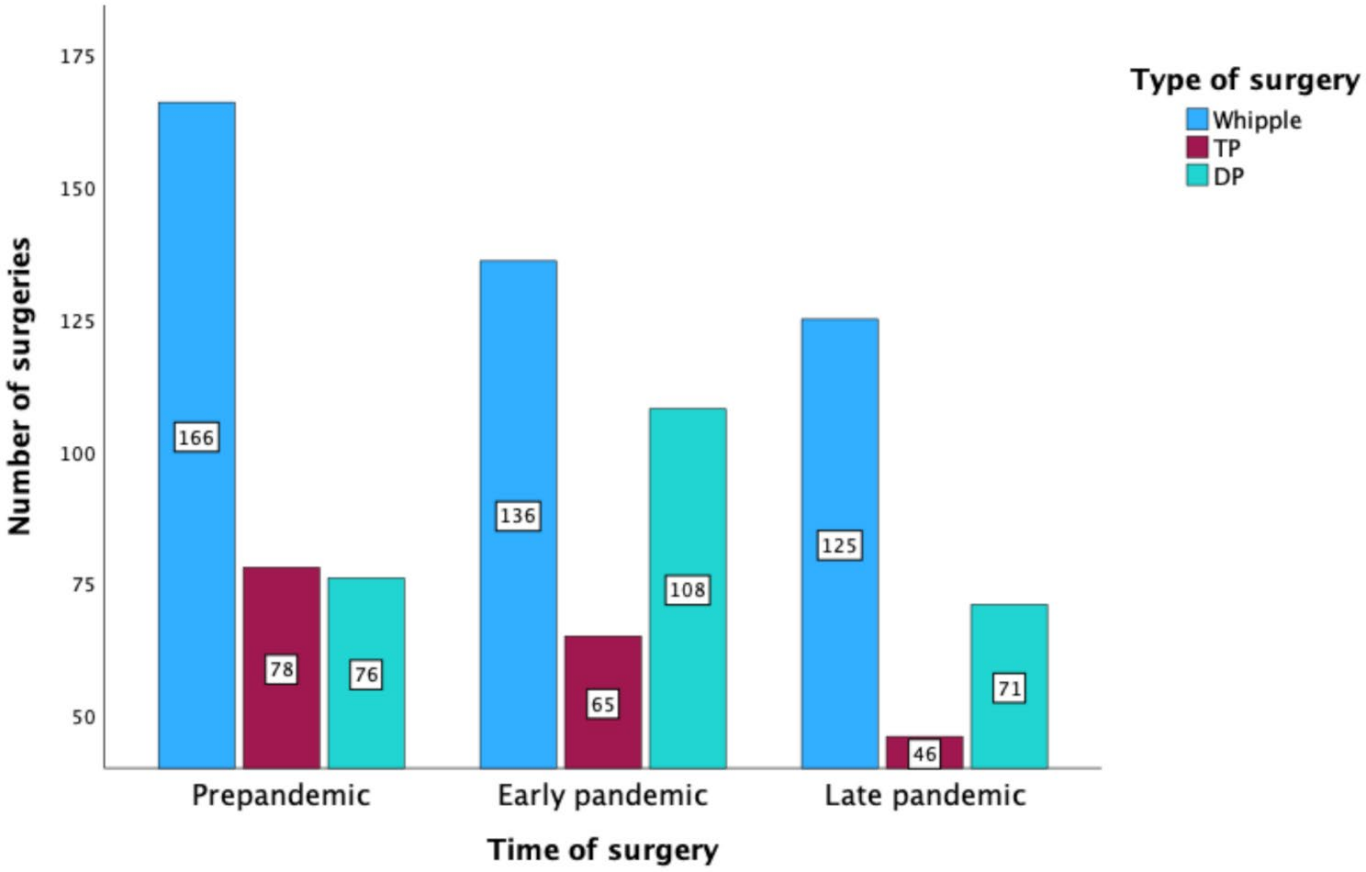
Number and type of pancreas surgeries performed in 2019–2022

Pre- and perioperative patient data are presented in Table 1. PDAC was the main indication for pancreas resection during all three periods. However, the rate of neoadjuvant therapy was significantly higher during the late pandemic period than during the early pandemic and pre-pandemic prior periods (*P =* 0.018). PD was the most common type of resection in all three periods, although fewer patients underwent PD during the early pandemic phase than during the other phases (Fig. 2). The complexity of PD was similar among the three periods (*P* = 0.207). TP procedures involved more multivisceral and arterial resections during the late pandemic phase, but were not significantly more complex (*P* = 0.077). In addition, the overall rate of surgical complications was higher during late pandemic phase (*P* = 0.043). POPF was the only postoperative complication that increased in frequency during the late pandemic phase, but this change was not significant (*P* = 0.085). The risk-adjusted rate of surgical complications, 30- and 90-day mortality are summarized in Table 2. There were no significant differences in the rate of mortality and the duration of ICU and hospital stay between the three periods.

**Table 2:** Risk-adjusted rates of surgical complications, 30-day and 90-day mortality after pancreatic surgery during the different timepoints

		Pre-pandemic phase	Early pandemic pahse	Late pandemic phase
Surgical complications	Observed rate	47.8%	45.6%	56.0%
	Risk-adjusted rate	**46.9%**	**46.9%**	**55.0%**
	95%CI	41.4–52.5	41.3–52.7	48.7–61.4
30-day mortality	Observed rate	1.6%	1.0%	3.3%
	Risk-adjusted rate	**1.2%**	**0.8%**	**2.5%**
	95%CI	0.07-2.0	0.0-1.7	0.5–4.5
90-day mortality	Observed rate	2.5%	1.6%	3.7%
	Risk-adjusted rate	**2.1%**	**1.4%**	**3.0%**
	95%CI	0.5–3.6	0.1–2.7	0.9–5.2

CI: confidence interval

### Patients with PDAC

The baseline characteristics and outcomes of patients diagnosed with PDAC are summarized in Table 3. The median age of patients was 67 years (range: 35–87) and 374 (47.3%) patients were male. The American Society of Anesthesiologists Physical Status (ASA) classification varied between the patients: 9.4% had an ASA I classification, 51.6% had an ASA II classification, and 39.1% had an ASA III classification. According to the preoperative radiological findings, the rate of primary resectable pancreatic tumors was significantly lower during the late pandemic phase, compared to pre- and early pandemic phases (67% vs. 66.9% vs. 56.5%, *P* = 0.024). Moderate to severe systemic disease was predominant among these patients during the early pandemic phase (*P* = 0.018). Only 221 (27.9%) patients with PDAC received neoadjuvant chemotherapy, without any significant difference in therapy regimen and number of cycles (*P* = 0.151 and *P* = 0.286). PD was the predominant surgery (49.3%), followed by DP (29.2%) and TP (21.5%). Notably, there were significantly more TP procedures during the early pandemic phase than during the pre-pandemic and late pandemic phases (*P* = 0.006). Morbidity was reported in 391 (49.4%) patients, and was significantly higher during the late pandemic period (*P* = 0.035). Postoperative complications such as DGE and PPH did not differ in patients with PDAC across the pandemic phases. The rate of POPF was 6.3% overall, and this rate increased slightly but not significantly to 9.6% in the late pandemic phase (*P* = 0.103). The overall 30-day mortality rate was 1.9% and the overall 90-day mortality rate was 2.5% and this did not change across the three time periods, despite the challenges presented by the pandemic (30-day mortality: *P* = 0.070, 90-day mortality: *P* = 0.192). The lengths of ICU, IMC, and hospital stays did not differ significantly among the pandemic phases, indicating consistent postoperative care for PDAC patients throughout the study period.

**Table 3:** Characteristics of patients undergoing pancreas surgery for pancreatic ductal adenocarcinoma in 2019–2022

		Total (*N* = 791)	Time			*P* value
			Pre-pandemic phase (*N* = 288)	Early pandemic phase (*N* = 287)	Late pandemic phase (*N* = 216)	
Age		67 (34–90)	67 (37–87)	69 (35–87)	67 (34–90)	0.217
Gender	Male	374 (47.3%)	132 (45.8%)	150 (52.3%)	92 (42.6%)	0.082
	Female	417 (52.7%)	156 (54.2%)	137 (47.7%)	124 (57.4%)	
ASA	I	74 (9.4%)	30 (10.4%)	19 (6.6%)	25 (11.6%)	**0.018**
	II	408 (51.6%)	159 (55.2%)	135 (47.0%)	114 (25.8%)	
	III	309 (39.1%)	99 (34.4%)	133 (46.3%)	77 (35.6%)	
Primary resectable tumor		507 (64.1%)	193 (67.0%)	192 (66.9%)	122 (56.5%)	**0.024**
Neoadjuvant chemotherapy		221 (27.9%)	75 (26.0%)	73 (25.4%)	73 (33.8%)	0.079
FOLFIRINOX		193 (87.3%)	66 (88.0%)	68 (93.2%)	59 (80.8%)	0.080
Gemcitabine		28 (12.7%)	9 (12.0%)	5 (6.8%)	14 (19.2%)	
Number of cycles		8 (2–28)	7 (3–12)	9 (2–18)	9 (2–28)	**0.009**
Procedure	PD	390 (49.3%)	151 (52.4%)	125 (43.6%)	114 (52.8%)	**0.006**
	TP	170 (21.5%)	72 (25.0%)	60 (20.9%)	38 (17.6%)	
	DP	231 (29.2%)	65 (22.6%)	102 (35.5%)	64 (29.6%)	
Procedure difficulty						
PD	Standard	208 (53.3%)	74 (49.0%)	69 (55.2%)	65 (57.0%)	0.158
	Venous resection	107 (27.4%)	48 (31.8%)	30 (24.0%)	29 (25.4%)	
	Multivisceral resection	56 (14.4%)	24 (15.9%)	15 (12.0%)	17 (14.9%)	
	Arterial resection	19 (4.9%)	5 (3.3%)	11 (8.8%)	3 (2.6%)	
TP	Standard	47 (27.6%)	17 (23.6%)	24 (40.0%)	6 (15.8%)	0.073
	Venous resection	38 (22.4%)	19 (26.4%)	10 (16.7%)	9 (23.7%)	
	Multivisceral resection	51 (30.0%)	23 (31.9%)	12 (20.0%)	16 (42.1%)	
	Arterial resection	34 (20.0%)	13 (18.1%)	14 (23.3%)	7 (18.4%)	
Operation time (min)		347.5 (110–877)	351 (117–640)	348 (110–877)	345 (117–662)	0.813
Blood loss (mL)		1100 (0–4800)	1100 (0–4800)	1000 (0–4700)	1100 (0–3800)	0.298
Morbidity		391 (49.4%)	139 (48.3%)	130 (45.3%)	122 (56.7%)	0.035
Clinically relevant DGE		28 (3.5%)	16 (5.6%)	7 (2.4%)	5 (2.3%)	0.067
Clinically relevant POPF*		39 (6.3%)	11 (5.1%)	11 (4.8%)	17 (9.6%)	0.103
Clinically relevant PPH		20 (2.5%)	10 (3.5%)	3 (1.0%)	7 (3.2%)	0.132
Relaparotomy		112 (14.4%)	45 (15.8%)	39 (13.7%)	28 (13.5%)	0.713
30-day mortality		15 (1.9%)	4 (1.4%)	3 (1.0%)	8 (3.7%)	0.070
90-day mortality		20 (2.5%)	6 (2.1%)	5 (1.7%)	9 (4.2%)	0.192
ICU stay		2 (1–82)	2 (1–82)	2 (1–60)	2 (1–30)	0.105
IMC stay		4 (1–87)	4 (1–83)	5 (1–87)	5 (2–42)	0.944
Hospital stay		14 (2–196)	14 (6–196)	14 (6–104)	15 (2–126)	0.366

Results are presented as median (range) or as *n* (%)

ASA: American Society of Anesthesiologists Physical Status; DGE: delayed gastric emptying; DP: distal pancreatectomy; ICU: intensive care unit; IMC: intermediate care; PD: pancreatoduodenectomy; POPF: postoperative pancreas fistula; PPH: postpancreatectomy hemorrhage; TP: total pancreatectomy

*Patients undergone TP were excluded from the analyses

### Different surgical procedures for PDAC

#### Whipple surgery

**Fig. 3:**
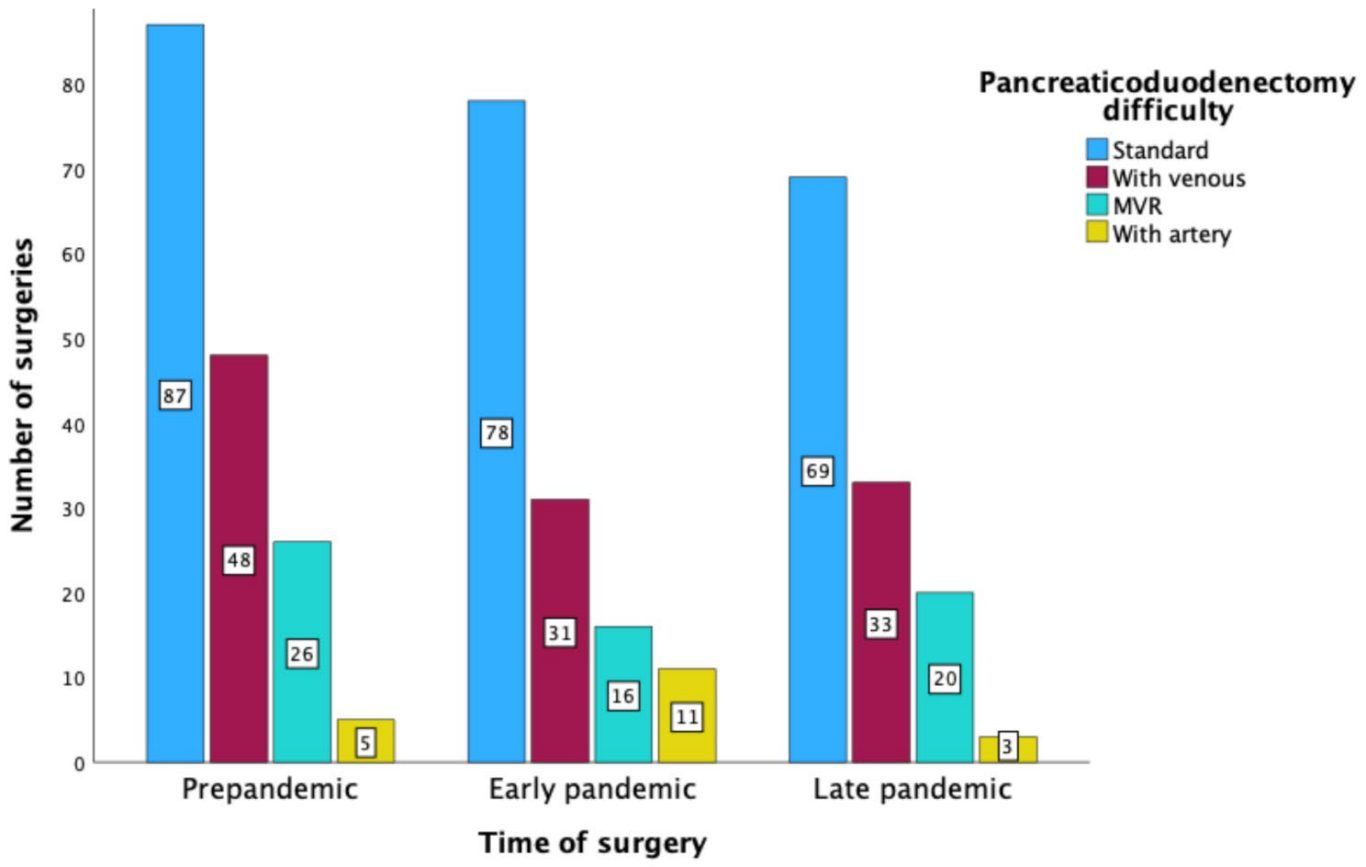
Difficulty of pancreaticoduodenectomy procedures performed in 2019–2022

During the study period, 390 patients underwent the Whipple procedure for PDAC. The demographic and clinical profile and the intraoperative and postoperative outcomes of these patients are detailed in Table 4. The median age of patients undergoing the Whipple procedure was 67 years (range: 38–90), and 56.4% were male. The age distribution and gender ratio of these patients did not differ significantly between the three study phases (age: *P* = 0.876, gender: *P* = 0.469). Of these patients, 24.9% received neoadjuvant chemotherapy prior to surgery, and there were no significant changes in preoperative treatment between phases (*P* = 0.217). The Whipple surgeries for PDAC were categorized based on complexity into standard PD (53.3%), PD with venous resection (27.4%), PD with multivisceral resection (14.4%), and PD with arterial resection (4.9%). As shown in Fig. 3, the complexity of surgery remained consistent across the study periods (*P* = 0.158). The median operation time was 365 min and the median blood loss was 1100 mL, with no significant differences between the three phases (operation time: *P* = 0.619, blood loss: *P* = 0.349). The overall morbidity was 52.3%, with no significant difference across the phases *(P* = 0.953); this high morbidity reflects the high complexity and risk associated with the Whipple procedure. Rates of clinically relevant DGE, POPF, and PPH were 4.1%, 5.6%, and 2.3% respectively, and were not significantly different between phases (DGE: *P* = 0.33, POPF: *P* = 0.25, and PPH: *P* = 0.815). The relaparotomy rate was 14.9% (*P* = 0.905), the 30-day mortality rate was 1.8%, and the 90-day mortality rate was 2.6%. No significant differences in mortality were observed between phases, except for a slight increase in 30-day mortality during the late pandemic phase (*P* = 0.046), which was not significant after post-hoc analysis. The median ICU stay was 2 days and the median hospital stay was 14 days, with no significant differences between pandemic phases (ICU stay: *P* = 0.360, hospital stay: *P* = 0.670).

**Table 4:** Characteristics of patients undergoing the Whipple procedure for pancreatic ductal adenocarcinoma in 2019–2022

		Total (*N* = 390)	Time			*P* value
			Pre-pandemic phase (*N* = 151)	Early pandemic phase (*N* = 125)	Late pandemic phase (*N* = 114)	
Age		67 (38–90)	68 (40–87)	67 (38–87)	66 (40–90)	0.876
Gender	Male	170 (43.6%)	71 (47.0%)	71 (56.8%)	45 (39.5%)	0.469
	Female	220 (56.4%)	80 (53.0%)	54 (43.2%)	69 (60.5%)	
ASA	I	74 (9.4%)	30 (10.4%)	19 (6.6%)	25 (11.6%)	**0.018**
	II	408 (51.6%)	159 (55.2%)	135 (47.0%)	114 (25.8%)	
	III	309 (39.1%)	99 (34.4%)	133 (46.3%)	77 (35.6%)	
Neoadjuvant chemotherapy		97 (24.9%)	31 (20.5%)	32 (25.6%)	34 (29.8%)	0.217
FOLFIRINOX		82 (84.5%)	26 (83.9%)	30 (93.8%)	26 (76.5%)	0.151
Gemcitabine		15 (15.5%)	5 (16.1%)	2 (6.3%)	8 (23.5%)	
Number of cycles		8 (2–20)	7.5 (3–15)	10 (2–18)	8 (2–20)	0.286
Operation time (min)		365 (110–735)	371 (174–590)	356 (110–735)	367 (147–662)	0.619
Blood loss (mL)		1100 (0–4800)	1050 (0–4600)	1100 (0–4500)	1000 (0–4800)	0.349
Morbidity		204 (52.3%)	78 (51.7%)	65 (52.0%)	61 (53.5%)	0.953
Clinically relevant DGE		16 (4.1%)	9 (6.0%)	4 (3.2%)	3 (2.6%)	0.331
Clinically relevant POPF*		22 (5.6%)	5 (3.3%)	8 (6.4%)	9 (7.9%)	0.251
Clinically relevant PPH		9 (2.3%)	4 (2.6%)	2 (1.6%)	3 (2.6%)	0.815
Relaparotomy		58 (14.9%)	22 (14.6%)	20 (16.0%)	16 (14.0%)	0.905
30-day mortality		7 (1.8%)	1 (0.7%)	1 (0.8%)	5 (4.4%)	**0.046**
90-day mortality		10 (2.6%)	3 (2.0%)	2 (1.6%)	5 (4.4%)	0.336
ICU stay		2 (1–31)	2 (1–26)	2 (1–31)	2 (1–25)	0.360
IMC stay		4 (1–47)	3 (1–47)	4 (2–31)	4 (2–22)	0.546
Hospital stay		14 (5–196)	14 (6–196)	13 (7–101)	14 (5–87)	0.670

Results are presented as median (range) or as *n* (%)

ASA: American Society of Anesthesiologists Physical Status; DGE: delayed gastric emptying; ICU: intensive care unit; IMC: intermediate care; POPF: postoperative pancreas fistula; PPH: postpancreatectomy hemorrhage

*Patients undergone TP were excluded from the analyses

#### Total pancreatectomy

**Fig. 4:**
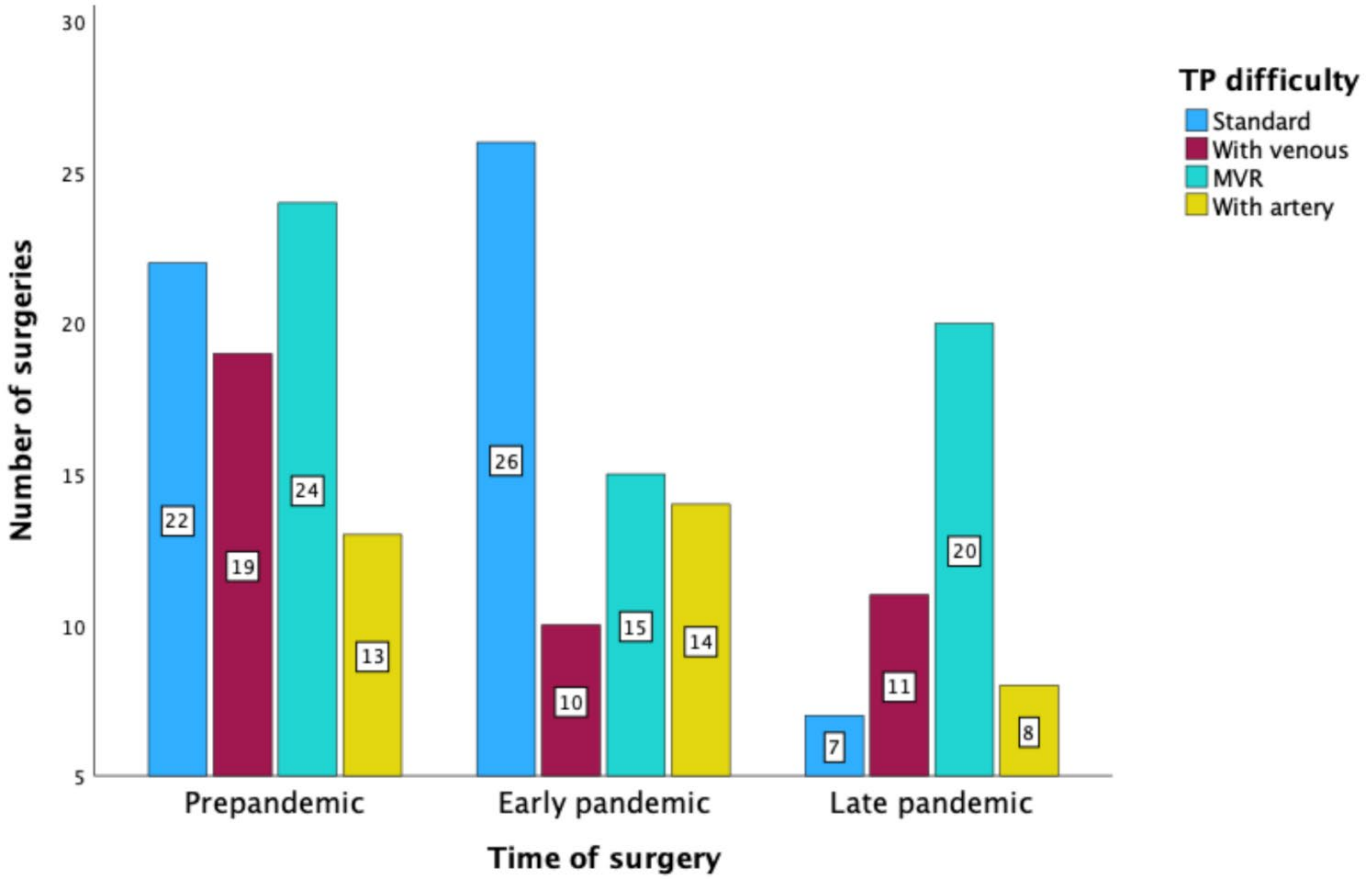
Difficulty of total pancreatectomy procedures performed in 2019–2022

The median age of patients undergoing TP was 67 years (range: 34–86). Notably, patients who underwent TP during the early pandemic phase were significantly older (median age: 69 years) than those who underwent TP during the pre-pandemic and late pandemic phase (*P* = 0.034). Gender was almost completely balanced in this cohort, with 50.6% of the patients being female (Table 5). Neoadjuvant chemotherapy was administered to 33.5% of patients, and was administered consistently across the pandemic phases (*P* = 0.874). The complexity of TP was categorized into standard TP (27.6%), TP with multivisceral resection (30.0%), TP with venous resection (22.4%), and TP with arterial resection (20.0%). As provided in Fig. 4, there was no significant difference in complexity across the pandemic phases (*P* = 0.073). The median operation time was 427 min and the median blood loss was 1700 mL, with no significant differences between study periods (operation time: *P* = 0.250, blood loss: *P* = 0.509). Postoperative morbidity was observed in 60.0% of cases and was significantly higher during the early pandemic phase (*P* = 0.004). The overall mortality rate was 4.7% after TP and did not differ significantly between the study periods. There were also no significant differences in the duration of ICU, IMC, and hospital stays between pandemic phases in this cohort.

**Table 5:** Characteristics of patients undergoing total pancreatectomy for pancreatic ductal adenocarcinoma in 2019–2022

		Total (*N* = 170)	Time			*P* value
			Pre-pandemic phase (*N* = 72)	Early pandemic phase (*N* = 60)	Late pandemic phase (*N* = 38)	
Age		67 (34–86)	66 (42–80)	69 (48–81)	65 (34–86)	0.135
Gender	Male	84 (49.4%)	31 (43.1%)	38 (63.3%)	17 (44.7%)	**0.048**
	Female	86 (50.6%)	41 (56.9%)	22 (36.7%)	21 (55.3%)	
ASA	I	11 (6.5%)	3 (4.2%)	4 (6.7%)	4 (10.5%)	0.208
	II	89 (52.4%)	33 (45.8%)	37 (61.7%)	19 (50.0%)	
	III	70 (41.2%)	36 (50.0%)	19 (31.7%)	15 (39.5%)	
Neoadjuvant chemotherapy		57 (33.5%)	23 (31.9%)	20 (33.3%)	14 (36.8%)	0.874
FOLFIRINOX		53 (93.0%)	22 (95.7%)	19 (95.0%)	12 (85.7%)	0.470
Gemcitabine		4 (7.0%)	1 (4.3%)	1 (5.0%)	2 (14.3%)	
Number of cycles		8 (2–15)	7 (3–15)	9 (2–14)	10 (3–15)	0.399
Operation time (min)		427 (200–877)	421 (234–640)	468 (200–877)	419 (247–662)	0.250
Blood loss (mL)		1700 (100–4800)	1650 (400–4700)	1850 (100–4500)	1900 (200–4800)	0.509
Morbidity		102 (60.0%)	37 (51.4%)	42 (70.0%)	23 (60.5%)	0.094
Clinically relevant DGE		5 (2.9%)	4 (5.6%)	1 (1.7%)	0 (0.0%)	0.200
Clinically relevant PPH		4 (2.4%)	3 (4.2%)	0 (0.0%)	1 (2.6%)	0.288
Relaparotomy		35 (20.6%)	17 (23.6%)	13 (21.7%)	5 (13.2%)	0.421
30-day mortality		6 (3.5%)	3 (4.2%)	2 (3.3%)	1 (2.6%)	0.913
90-day mortality		7 (4.1%)	3 (4.2%)	3 (5.0%)	1 (2.6%)	0.847
ICU stay		2 (1–82)	2 (1–82)	2.5 (1–16)	2 (1–7)	0.773
IMC stay		5 (1–87)	5 (1–83)	5 (2–87)	5 (2–20)	0.377
Hospital stay		18 (2–139)	17 (7–139)	19 (10–104)	17 (2–49)	0.673

Results are presented as median (range) or as *n* (%)

ASA: American Society of Anesthesiologists Physical Status; DGE: delayed gastric emptying; ICU: intensive care unit; IMC: intermediate care; PPH: postpancreatectomy hemorrhage

#### Distal pancreatectomy

As shown in Table 6, median age of patients undergoing DP was 68 years (range: 35–89). Patient age did not differ significantly between the pandemic phases (*P* = 0.387). Gender distribution was nearly even, with 51.5% of patients being male, and there was no significant variation in gender between groups (*P* = 0.294). Notably, neoadjuvant chemotherapy was administered to 29.0% of patients overall, and this increased significantly to 39.1% in the late pandemic phase (*P* = 0.030). Besides, patients who underwent TP during the late pandemic phase received a significantly higher number of neoadjuvant therapy cycles (*P* = 0.002). The median operation time for DP was 235 min, and this increased slightly to 269 min in the late pandemic phase (*P* = 0.012). The median blood loss was 700 mL, and did not differ significantly between study periods (*P* = 0.778). Morbidity was observed in 37.0% of patients overall, and this increased significantly to 60.3% in the late pandemic phase (*P* < 0.001). The rates of postoperative DGE and PPH were both 3.0% and remained consistent across phases (DGE: *P* = 0.620, PPH: *P* = 0.271). The rate of relaparotomy was 8.2% overall and did not differ significantly between study phases (*P* = 0.484). The 90-day mortality rate was 1.3% overall, with significantly highest rate of 4.7% in the late pandemic phase (*P* = 0.019). The median ICU stays was 2 days, and increased slightly during the late pandemic phase (*P* = 0.032). The length of hospital stay also increased marginally during the late pandemic phase (median: 14.5 days, *P* = 0.041).

**Table 6:** Characteristics of patients undergoing distal pancreatectomy for pancreatic ductal adenocarcinoma in 2019–2022

		Total (*N* = 231)	Time			*P* value
			Pre-pandemic phase (*N* = 65)	Early pandemic phase (*N* = 102)	Late pandemic phase (*N* = 64)	
Age		68 (35–89)	65 (37–87)	72 (35–84)	68 (43–89)	0.387
Gender	Male	118 (51.1%)	30 (46.2%)	58 (56.9%)	30 (46.9%)	0.294
	Female	113 (48.9%)	35 (53.8%)	44 (43.1%)	34 (53.1%)	
ASA	I	25 (10.8%)	6 (9.2%)	10 (9.8%)	9 (14.1%)	0.852
	II	109 (47.2%)	31 (47.7%)	47 (46.1%)	31 (48.4%)	
	III	97 (42.0%)	28 (43.1%)	45 (44.1%)	24 (37.5%)	
Neoadjuvant chemotherapy		67 (29.0%)	21 (32.3%)	21 (20.6%)	25 (39.1%)	**0.030**
FOLFIRINOX		58 (86.6%)	18 (85.7%)	19 (90.5%)	21 (84.0%)	0.806
Gemcitabine		9 (13.4%)	3 (14.3%)	2 (9.5%)	4 (16.0%)	
Number of cycles		8 (3–28)	9 (3–14)	9 (3–14)	10 (6–28)	**0.002**
Operation time (min)		235 (117–540)	215 (117–510)	233 (125–510)	269 (117–540)	**0.012**
Blood loss (mL)		700 (20–4000)	700 (20–2700)	700 (100–4000)	800 (50–2800)	0.778
Morbidity		85 (37.0%)	24 (36.9%)	23 (22.5%)	38 (60.3%)	**< 0.001**
Clinically relevant DGE		7 (3.0%)	3 (4.6%)	2 (2.0%)	2 (3.1%)	0.620
Clinically relevant POPF		17 (7.4%)	6 (9.2%)	3 (2.9%)	8 (12.5%)	0.057
Clinically relevant PPH		7 (3.0%)	3 (4.6%)	1 (1.0%)	3 (4.7%)	0.271
Relaparotomy		19 (8.2%)	6 (9.2%)	6 (5.9%)	7 (10.9%)	0.484
30-day mortality		2 (0.9%)	0 (0.0%)	0 (0.0%)	2 (3.1%)	0.072
90-day mortality		3 (1.3%)	0 (0.0%)	0 (0.0%)	3 (4.7%)	**0.019**
ICU stay		2 (1–60)	2 (1–24)	1 (1–60)	2 (1–30)	**0.032**
IMC stay		5.5 (1–56)	5.5 (2–56)	3 (1–17)	5 (2–42)	0.125
Hospital stay		13 (7–128)	11 (6–126)	12 (6–101)	14.5 (6–126)	**0.041**

Results are presented as median (range) or as *n* (%)

ASA: American Society of Anesthesiologists Physical Status; DGE: delayed gastric emptying; ICU: intensive care unit; IMC: intermediate care; POPF: postoperative pancreas fistula; PPH: postpancreatectomy hemorrhage

## Discussion

Pancreatic cancer (predominantly PDAC) is the fourth leading cause of cancer-related death worldwide. Because the symptoms are non-specific, diagnosis and treatment are often delayed until surgical treatment is the only feasible approach to curing the cancer and increasing the overall survival of patients. Therefore, timely access to surgical therapy is crucial for effective, good-quality care. Unfortunately, access to surgical treatment was disrupted by the COVID-19 pandemic.

In 2020, there was a state-wide lockdown in Germany [18, 19], and the high number of patients with severe COVID-19 put excess pressure on the health care system [20, 21]. Elective surgeries were cancelled or postponed, and only emergency operations were performed [21, 22]. In tertiary surgical centers, ICU and IMC beds were restricted by the high number of patients with COVID-19-related respiratory conditions [23]. This also affected our center, which is a referral center for HPB surgery. During the lockdown, most of our intensive care services were dedicated to patients with COVID-19, which reduced our capacity to deliver postoperative care to patients undergoing elective HPB surgeries [24–27]. In the early phase of the pandemic, cancer was classified as an emergency, so treatment was not delayed. In terms of multidisciplinary management, the interdisciplinary tumor board successfully adapted to the restrictions imposed by the pandemic by initiating online sessions. This approach prevented delays in the decision-making process. However, unlike the findings by Javed et al. [28], local medical imaging facilities were impacted by the pandemic, resulting in reduced diagnostic capacity. To mitigate this, patients were encouraged to undergo diagnostic procedures at our institution, which helped minimize waiting times. An additional advantage was the improved quality of imaging achieved through standardized diagnostic protocols. Moreover, the imaging findings were reviewed preoperatively by HPB-expert radiologists and shared with the responsible surgeons, facilitating accurate and precise surgical planning. Also, we took certain measures to ensure that patients with PDAC received the timely surgical treatment they needed to increase survival. First, we prioritized candidates for up-front surgery and high-risk patients for pancreas resection. By postponing non-oncological elective operations, we were able to perform pancreas resection with oncological indications. We applied strict and rigorous SARS-CoV-2 screening to prevent in-hospital outbreaks and postponed operations if the patient had a confirmed infection. To reduce burden on postoperative care units, only patients who underwent complex surgery or had a complicated intraoperative course were admitted to the IMC and ICU. Patients with uncomplicated or standard PD and DP were transferred to normal wards after a short monitoring in the postoperative care unit. We implemented these measures from the start of lockdown until the vaccination campaign was introduced.

These measures ensured that the number of pancreas surgeries in our center, especially for patients with PDAC, did not decrease during the pandemic (Fig. 1). Indeed, the number of procedures we performed even increased by 5% during the early pandemic phase and remained steady throughout the pandemic. The low number of surgeries performed in January and April 2020 (Fig. 1) cannot necessarily be considered due to the pandemic considering the overall picture. The center management also increased the number of pancreas resections that were carried out daily to prevent further delays, which explains the peak numbers in September–December 2020. We also received referrals for pancreas resection from other centers who were overwhelmed by patients with COVID-19. Taken together, these elements increased the number of pancreas resections performed at our center during the pandemic and ensured that the waiting list for pancreas resection was not extended. Also of note, the number of patients undergone neoadjuvant chemotherapies was significantly higher in the late pandemic phase than during the early and pre-pandemic phases. This is because neoadjuvant therapy was indicated due to delayed surgery in delayed in some patients with PDAC in the early pandemic phase and then rescheduled with surgical treatment later on. The consensus on the management of pancreatic surgery during the COVID-19 pandemic recommended the use of neoadjuvant therapy for patients with non-metastatic disease, regardless of the stage of the disease [8]. However, during the study period—particularly in the early phase of the pandemic—the majority of patients presented with primarily resectable disease and underwent timely surgical resection without unnecessary delays caused by neoadjuvant therapy. This highlights the effectiveness of management adaptations implemented at a high-volume HPB center in mitigating the impact of the pandemic. Regarding neoadjuvant therapy, its duration was extended during the later phase of the pandemic. This approach aligns with the recommendations of Oba et al. [8], who advocated for prolonged neoadjuvant therapy in situations with limited healthcare resources.

Our finding that very few surgeries were postponed in our department during the pandemic and that the waiting list was not extended is in contrast to reports from the COVIDSurg Collaborative of a median 23-week delay for oncological surgeries [2]. Likewise, Marchegiani et al. reported a 20% reduction in the number of pancreas resections in 2020, particularly between March and May [29]. Their lowest number of resections was reported in April 2020, which is similar to our results. This reduction in the number of surgeries during the early phase of the pandemic should not be overlooked [30], despite our promising finding that the number of surgeries then increased and stayed constant throughout the pandemic. In agreement with our findings, Hanssen et al., also showed no significant reduction in the treatment of malignant pancreatic and periampullary tumors during the pandemic [31], with the number of operations at their center staying similar to those preceding the pandemic.

Compared to previous studies [8, 29], our adaptations during the COVID-19 pandemic not only maintained the surgical volume for pancreatic cancer surgery at our center but also avoided the unnecessary administration of neoadjuvant treatments in patients with primarily resectable tumors. Furthermore, our measures facilitated an increase in the number of pancreatic cancer surgeries through the centralization of care. This was particularly impactful at a time when less experienced centers were unable to perform surgical treatments for pancreatic cancer. We also examined the effect of the pandemic on the outcomes of pancreatic resection and found no changes in intraoperative outcomes throughout the pandemic. Significantly fewer TPs were performed during the early phase of the pandemic, but no changes in the complexity and difficulty of PD and TP were observed during the pandemic. We looked at outcomes in patients with PDAC following pancreas resection and found no significant differences in intraoperative and mortality rates before and during the pandemic, except in postoperative morbidity. Subgroup analyses revealed significantly higher rates of morbidity and mortality in patients who underwent DP during the late pandemic phase. This likely reflects locally advanced PDAC in these patients caused by delayed neoadjuvant chemotherapy, which contraindicated up-front surgery and complicated the surgical procedure. We also observed a higher rate of POPF; this might not have been correlated to the pandemic but rather to patient characteristics and complexity of the procedure. These findings suggest that our approach of prioritizing pancreas surgery with oncological indications maintained not only good surgical frequency but also good surgical quality during the challenges of the pandemic.

There are some limitations to this study. First, this was a descriptive analysis of patient outcomes in a highly specialized tertiary surgical center and the results may not be generalizable to other surgical environments. In addition, we did not evaluate the effect of neoadjuvant and adjuvant therapies or their delay on patient outcomes during the pandemic. Another limitation is that we only examined the short-term effects of the pandemic. Longer follow ups are needed to determine the long-term effects of the pandemic on oncological outcomes. Future studies should also look at the effects of the pandemic on other important aspects, such as surgical education and academic research.

In conclusion, the COVID-19 pandemic did not disrupt oncological pancreas resection at our center. Our findings show that centralizing pancreas surgery with a standardized postoperative approach can overcome the challenges of a pandemic in a high-volume and highly specialized surgical center. This approach may improve resistance to unexpected events in the future.

## Data Availability

The datasets used and/or analyzed during the current study are available from the corresponding author on reasonable request.
